# Vitamin D Treatment Prevents Uremia-Induced Reductions in Aortic microRNA-145 Attenuating Osteogenic Differentiation despite Hyperphosphatemia

**DOI:** 10.3390/nu14132589

**Published:** 2022-06-22

**Authors:** Natalia Carrillo-López, Sara Panizo, Maria Vittoria Arcidiacono, Sandra de la Fuente, Laura Martínez-Arias, Emerenziana Ottaviano, Catalina Ulloa, María Piedad Ruiz-Torres, Isabel Rodríguez, Jorge B. Cannata-Andía, Manuel Naves-Díaz, Adriana S. Dusso

**Affiliations:** 1 Bone and Mineral Research Unit, Hospital Universitario Central de Asturias, Instituto de Investigación Sanitaria del Principado de Asturias (ISPA), 33011 Oviedo, Spain; ncarrillolopez.huca@gmail.com (N.C.-L.); sarapanizogarcia@gmail.com (S.P.); lauramartinezarias@gmail.com (L.M.-A.); emerenziana.ottaviano@gmail.com (E.O.); catalinaulloac@hotmail.com (C.U.); isabelrodriguez2710@gmail.com (I.R.); mnaves.huca@gmail.com (M.N.-D.); 2 Redes de Investigación Cooperativa Orientadas a Resultados en Salud (RICORS), RICORS2040 (Kidney Disease), 28040 Madrid, Spain; 3 Division of Experimental Nephrology, IRB Lleida, 25198 Lleida, Spain; arcivicki@hotmail.com (M.V.A.); sandra.fuente.ruiz@gmail.com (S.d.l.F.); 4 Departamento de Biología de Sistemas, Universidad de Alcalá, 28801 Alcalá de Henares, Spain; mpiedad.ruiz@uah.es; 5 Departamento de Medicina, Universidad de Oviedo, 33006 Oviedo, Spain; 6 Division of Endocrinology, Metabolism and Lipid Research, Washington University School of Medicine, St. Louis, MO 63110, USA

**Keywords:** vascular injury, osterix, α-actin, runx2, osteogenic differentiation, vitamin D

## Abstract

In chronic kidney disease, systemic inflammation and high serum phosphate (P) promote the de-differentiation of vascular smooth muscle cells (VSMC) to osteoblast-like cells, increasing the propensity for medial calcification and cardiovascular mortality. Vascular microRNA-145 (miR-145) content is essential to maintain VSMC contractile phenotype. Because vitamin D induces aortic miR-145, uremia and high serum P reduce it and miR-145 directly targets osteogenic osterix in osteoblasts, this study evaluated a potential causal link between vascular miR-145 reductions and osterix-driven osteogenic differentiation and its counter-regulation by vitamin D. Studies in aortic rings from normal rats and in the rat aortic VSMC line A7r5 exposed to calcifying conditions corroborated that miR-145 reductions were associated with decreases in contractile markers and increases in osteogenic differentiation and calcium (Ca) deposition. Furthermore, miR-145 silencing enhanced Ca deposition in A7r5 cells exposed to calcifying conditions, while miR-145 overexpression attenuated it, partly through increasing α-actin levels and reducing osterix-driven osteogenic differentiation. In mice, 14 weeks after the induction of renal mass reduction, both aortic miR-145 and α-actin mRNA decreased by 80% without significant elevations in osterix or Ca deposition. Vitamin D treatment from week 8 to 14 fully prevented the reductions in aortic miR-145 and attenuated by 50% the decreases in α-actin, despite uremia-induced hyperphosphatemia. In conclusion, vitamin D was able to prevent the reductions in aortic miR-145 and α-actin content induced by uremia, reducing the alterations in vascular contractility and osteogenic differentiation despite hyperphosphatemia.

## 1. Introduction

In Chronic Kidney Disease (CKD), the progressive decreases in serum 25-hydroxyvitamin D_3_ (25(OH)D_3_) and 1α,25-dihydroxyvitamin D_3_ (1,25D_3_) levels increase the propensity for vascular calcification (VC), arterial stiffness and heart failure, increasing the risk for cardiovascular mortality [1,2,3,4,5,6].

One important effect of 1,25D_3_ is the induction of microRNA-145 (miR-145) gene expression [7], a prevalent miR in vascular smooth muscle cells (VSMC), which is the master regulator of VSMC fate and could provide protection against CKD-induced vascular injury [8]. 

Reductions in VSMC miR-145 content facilitate the loss of their contractile phenotype and an inappropriate de-differentiation towards a higher proliferative, migratory or synthetic phenotype, responsible for blunted hypertensive responses to Angiotensin II, arterial thickening, restenosis and atherosclerosis [9,10,11,12]. Therefore, in CKD, vitamin D deficiency could aggravate the VSMC injury associated with miR-145 reduction.

Hyperphosphatemia, a recognized risk factor for VC and cardiovascular mortality, even in individuals with normal renal function [13], also reduces the miR-145 content of normal aortas [14]. Similar to what has been described for miR-125b, the first microRNA directly associated with human artery calcification, miR-145 down-regulates the expression of osteogenic genes in osteoblasts [15,16] and directly targets the transcription factor krüppel-like factor 4 (KLF4), which also controls osteogenic gene expression [17,18,19]. Furthermore, miR-145 overexpression increases, and its silencing decreases the expression of α-actin and other markers of the integrity of the arterial elastic layer. Therefore, it is possible that in CKD, both hyperphosphatemia and 25(OH)D_3_/1,25D_3_ deficiency aggravate the uremia-induced miR-145 reductions, favoring VSMC de-differentiation towards osteogenic-driven medial calcification.

This study was designed (a) to evaluate the efficacy of vitamin D in the prevention of aortic miR-145 reduction and its associated vascular injury; and (b) to evaluate the potential links between the reduction in miR-145, osterix/Runx2-driven osteogenic differentiation and calcium (Ca) deposition in VSMC.

## 2. Materials and Methods

### 2.1. General Design of the Study

To achieve the objectives of the study, two different approaches were followed: (A) In vivo: To investigate, in a mouse model of mild Chronic Renal Failure (CRF) the efficacy of the vitamin D system in the prevention of aortic miR-145 reduction and its associated vascular injury. (B) Ex vivo and in vitro: To investigate in aortic rings and VSMC the potential links between the reduction in miR-145, osterix/Runx2-driven osteogenic differentiation and Ca deposition.

### 2.2. In Vivo Study

#### 2.2.1. Experimental Design

Nine-week-old FVB/N mice, a strain chosen for its higher susceptibility to develop secondary hyperparathyroidism by 2 months after nephrectomy (NX), underwent a two-step 75% reduction in renal mass [20]. Briefly, upon de-capsulation of the left kidney, upper and lower poles were removed and one week later, the right kidney was removed. Mice were fed a normal chow diet (Envigo, Indianapolis, IN, USA) and two months after NX, mice with a similar degree of renal damage (measured by blood urea nitrogen (BUN)) were re-grouped to receive for 6 additional weeks either vehicle (*n* = 10) or 25(OH)D_3_ (i.p., 80 ng weekly; Sigma-Aldrich, St. Louis, MO, USA) + 19-nor-1α,25-dihydroxyvitamin D2 (paricalcitol, kindly provided by Abbot Pharmaceuticals (Chicago, IL, USA), i.p., 1.6 ng thrice weekly; diluted in 50 µL of propylenglycol (*n* = 10)). The doses of 25(OH)D_3_ and paricalcitol used were estimated from a previous study performed in a hyperphosphatemic rat CKD model [21] and they were adjusted considering a metabolic mouse:rat ratio of 2:1. This combination efficaciously corrects both vitamin D deficiency and secondary hyperparathyroidism in uremic rats, with the serum Ca and P levels remaining unchanged, results not achievable using monotherapy with the same dosage of either vitamin D or paricalcitol [21]. 

A sham group fed the same diet served as a negative control of renal and aortic damage (*n* = 10). At sacrifice (14 weeks later), blood was collected and sections from kidneys and thoraco-abdominal aortas were either frozen for total RNA and total Ca measurements, or processed for immunohistochemical of renal ADAM17 analyses, as a markers of renal and vascular damage [22,23].

Approval for the animal study was obtained from the Ethics Committee for Animal Experimentation at Lleida University in compliance with current international legislation for animal research.

#### 2.2.2. Blood Chemistries

QuantiChrom™ Urea Assay Kit was used for BUN (DIUR-100, Bioassay Systems, Hayward, CA, USA), QuantiChrom™ Calcium Assay Kit for plasma Ca (DICA-500, Bioassay Systems), QuantiChrom™ Phosphate Assay Kit for plasma P (DIPI-500, BioAssay Systems), mouse intact PTH ELISA Kit for intact parathyroid hormone (PTH, Immutopics, San Diego, CA, USA) and the mouse C-terminal FGF23 ELISA for fibroblast growth factor 23 (FGF23, EMD Millipore, Burlington, VT, USA).

#### 2.2.3. Immunofluorescence

Kidney sections (5 µm) were deparaffinized and rehydrated. Tissue autofluorescence was attenuated by 2h-exposure to UV light in PBS, followed by 20 min of blocking in 0.1% Sudan black solution. Sections were then blocked with 3% BSA in PBS and incubated overnight at 4 °C with anti-ADAM17 antibody (1:100, Millipore, Burlington, VT, USA). Next, anti-rabbit Alexa Fluor 488 was added for 1h at RT. Nuclear counterstaining was performed using Hoescht (1:500, Invitrogen, Waltham, MA, USA). Ten different sections were measured for each sample. The measurements were blinded, and semiautomatic image analysis software (Image J software) was used. A negative control without primary antibody was used to set the level of the lowest detectable staining intensity.

#### 2.2.4. Alizarin Red Staining

Five µm paraffin sections of mouse aortas were deparaffinized, rehydrated and stained with red Alizarin to estimate Ca deposition [24].

### 2.3. Ex Vivo and In Vitro Studies

#### 2.3.1. Experimental Design

For ex vivo studies, aortic rings (1–2 mm) from normal rats, washed in cold PBS containing P/S, were placed in fibronectin pre-coated (100 µg/mL) 6-well plates (8 rings/well) containing growing media.

A7r5 cells (a rat aortic VSMC cell line) (ATCC) were grown in 10% FBS DMEM (Lonza, Basilea, Switzerland) containing 2 mM L-glutamine, 1 g/L D-glucose, 3.7 g/L sodium bicarbonate and 100 U/mL penicillin/100 μg/mL streptomycin (P/S) at 37 °C, in a humidified incubator with 5% CO2.

A7r5 cells and aortic rings were incubated for 4 days, either in a non-calcifying medium (Non-CM: DMEM-F12 + 0.1% BSA containing 1 mM Ca, 1 mM phosphorus (P)) or in a calcifying medium (CM: DMEM-F12 + 0.1% BSA containing 2 mM Ca, 3 mM P).

#### 2.3.2. Transfection with Antagomirs and Mimics

A7r5 cells were seeded at a concentration of 1 × 10^5^ cells per well in 6-well plates to reach overnight 60–70% confluency. Afterwards, they were transfected with the DharmaFECT transfection reagent (GE Healthcare Dharmacon, Lafayette, LA, USA) with 500 pmol of miR-145 mimic (for miR-145 overexpression), an antisense oligonucleotide of miR-145 (antagomir, to inhibit miR-145 expression), or scrambled sequence (as negative miRNA control; Thermo-Fisher Scientific, Waltham, MA, USA), following manufacturer’s instructions. Transfected cells were incubated for 4 days as indicated above. Cells were then collected for total Ca measurement and gene expression analysis.

### 2.4. Common Analytical and Technical Procedures Used in All the Studies

#### 2.4.1. Total Calcium Measurement

To measure Ca concentration in the three models, a 20 mm segment of the mouse aortas, rat aortic rings and A7r5 cells were used. They were washed thricely with PBS and decalcified with 0.6 N HCl. After a 24 h-shaking at 4 °C, the samples were centrifuged. Total Ca was measured in supernatants by o-cresolphthalein-complexone [25] and normalized by total RNA (for aortic tissues) or protein content (for A7r5 cells).

#### 2.4.2. Quantitative PCR

The total RNA from mouse aortas, rat aortic rings and A7r5 cells, was extracted using TRI reagent (Sigma-Aldrich, St. Louis, MO, USA). After reverse transcription using a High-Capacity cDNA Reverse Transcription Kit (Applied Biosystems, Waltham, MA, USA), quantitative-real time PCR (qPCR) reactions were performed in triplicate using the Stratagene Mx3005P QPCR System (Agilent Technologies, Santa Clara, CA, USA), Fast Start Universal Probe Master (Roche) and pre-developed assays (Thermo-Fisher Scientific, Waltham, MA, USA). Quantification of target genes relative to GAPDH or U6 gene expression was performed by comparing threshold cycles using the ΔΔCT method [26].

#### 2.4.3. Statistical Analysis

Data are shown as Mean ± SD or medians and interquartile ranges according to the normal or non-normal distribution of the examined variable. Accordingly, parametric (Student *t*-test) or non-parametric tests (Mann–Whitney U test or Dunn test for multiple comparisons) were used to evaluate the statistical differences between groups using SPSS 17.0 or R for Windows.

## 3. Results

### 3.1. In Vivo Study

#### 3.1.1. Biochemical Parameters and Kidney Function

**Figure 1:**
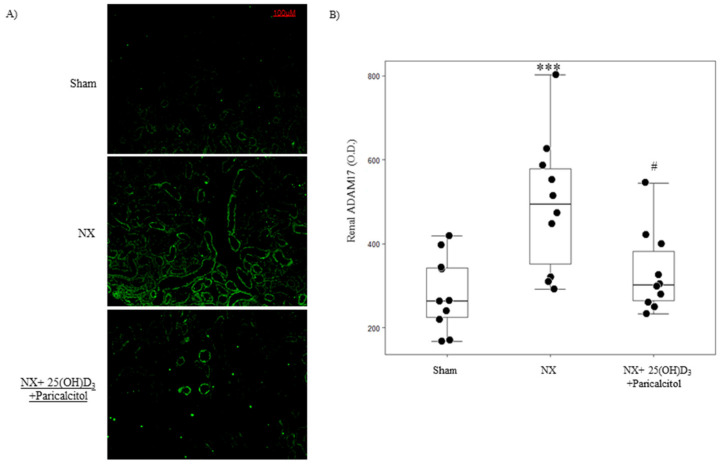
Vitamin D prevents uremia-induced increases in renal ADAM17 protein. (**A**) Representative images of ADAM17 immunofluorescence and (**B**) and its quantification in kidneys from mice subjected to 75% NX and exposed to vehicle (NX group, *n* = 10) or to the combination of 25(OH)D_3_ and paricalcitol (25(OH)D_3_: 80 ng/mice i.p. weekly and Paricalcitol: 2.6 ng/mice i.p. thrice weekly; NX + 25(OH)D_3_ + Paricalcitol, *n* = 10) for 6 weeks, after 8 weeks of uremia in mice fed a normal diet. A group with normal renal function was used as control (Sham group, *n* = 10). Box plots represent median and interquartile range; *** *p* < 0.001 vs. Sham and # *p* < 0.05 vs. NX group.

Fourteen weeks after the induction of the 75% nephrectomy (NX group), 2-fold elevation in serum BUN and PTH levels with no significant increases in serum Ca, P or C-terminal FGF23 levels were observed (Table 1). The combination of 25(OH)D_3_ and paricalcitol for 6 weeks did not change BUN levels compared to the Sham and NX groups, but the group that received the vitamin D combined therapy showed a significant increase in serum Ca, P and FGF23 levels and a significant decrease in serum PTH levels (Table 1). In addition, NX mice showed a significant increase in renal ADAM17 protein expression, which was prevented with the use of the combination of 25(OH)D_3_ and paricalcitol (Figure 1).

#### 3.1.2. Aortic Osteogenic Differentiation and Calcification

**Figure 2:**
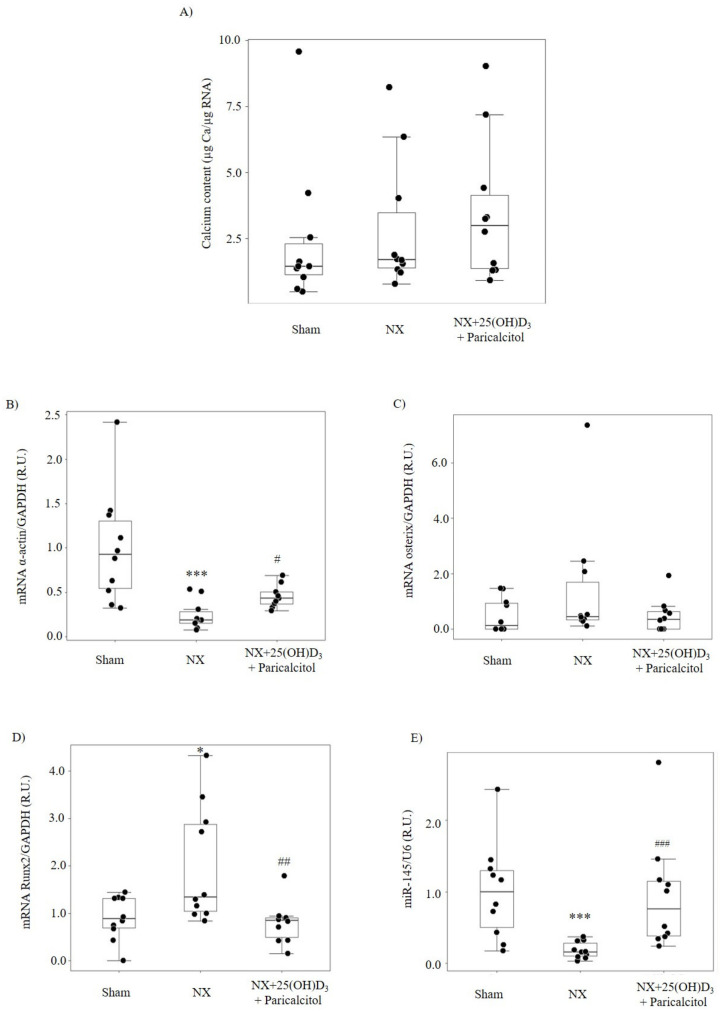
Vitamin D prevents uremia-induced aortic osteogenic differentiation and calcification. (**A**) Ca content, (**B**) α–actin, (**C**) osterix, (**D**) Runx2 and (**E**) miR-145 levels in aortas from mice subjected to 75% NX and exposed to vehicle (NX group, *n* = 10) or to the combination of 25(OH)D_3_ and paricalcitol (25(OH)D_3_: 80 ng/mice i.p. weekly and Paricalcitol: 2.6 ng/mice i.p. thrice weekly; NX + 25(OH)D_3_ + Paricalcitol, *n* = 10) for 6 weeks, after 8 weeks of uremia in mice fed a normal diet. A group with normal renal function was used as control (Sham group, *n* = 10). Bars and error bars represent mean ± SD and box plots represent median and interquartile range; R.U.: relative units. * *p* < 0.05 and *** *p* < 0.001 vs. Sham and # *p* < 0.05, ## *p* < 0.01 and ### *p* < 0.001 vs. NX group.

In the NX group, no significant changes in aortic Ca content were observed compared with the Sham group and the Alizarin red staining was negative (not shown) (Figure 2A). A 77% reduction in aortic α-actin mRNA and non-significant elevation in aortic osterix mRNA levels were observed (Figure 2B,C). By contrast, aortic Runx2 mRNA was significantly higher compared to sham (Figure 2D) and aortic miR-145 expression decreased by 85% (Figure 2E).

In the group that received 25(OH)D_3_ and paricalcitol, no significant changes in Ca content were observed (Figure 2A), but both aortic α-actin mRNA and miR-145 expression were significantly higher compared to the NX group (Figure 2B,E). No changes in Osterix (Figure 2C) and significant lower Runx2 mRNA levels (Figure 2D) were observed compared to the NX group, with similar values to those observed in the sham group.

### 3.2. Ex Vivo and In Vitro Studies

#### 3.2.1. Effect of Calcifying Medium on Aortic Rings and VSMC Osteogenic Differentiation and Calcification

**Figure 3:**
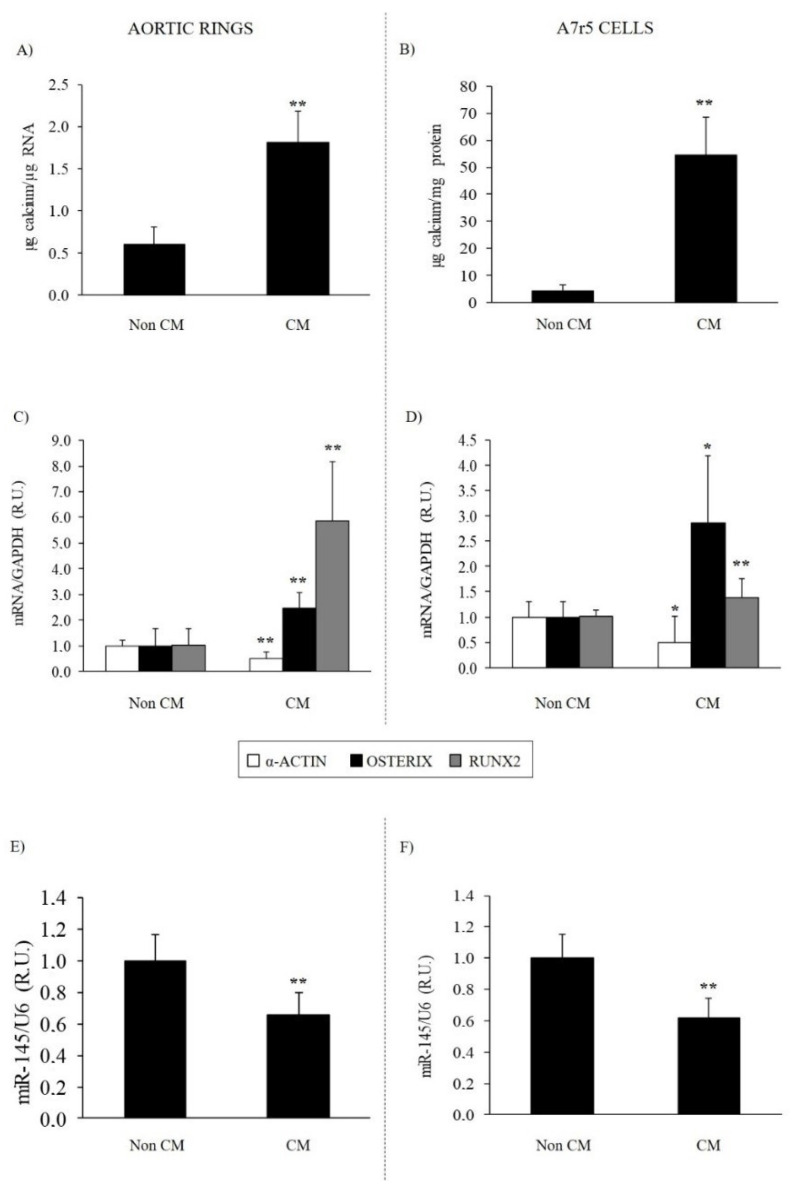
Calcification and osteogenic differentiation in aortic rings and VSMC under calcifying conditions. Ca deposition in aortic rings (**A**) and A7r5 cells (**B**) exposed to non-calcifying media (Non CM: 1 mM Ca; 1 mM P) or calcifying media (CM: 2mM Ca; 3mM P) for 4 days. Gene expression of α–actin (white bars), osterix (black bars) and Runx2 (gray bars) in aortic rings (**C**) and A7r5 cells (**D**) treated as described. Levels of miR-145 in aortic rings (**E**) and in A7r5 cells (**F**) treated as described. Bars and error bars represent mean ± SD from three independent experiments performed in triplicate per experimental condition. R.U.: relative units. * *p* < 0.05 and ** *p* < 0.01 vs. non-CM.

The exposure of aortic rings from normal rats or A7r5 cells to CM for 4 days increased total Ca deposition by 3- and 13-fold, respectively (Figure 3A,B). The CM significantly reduced α-actin mRNA by 51% and miR-145 by 40% in aortic rings and α-actin mRNA by 52% and miR-145 by 40% in A7r5 cells (Figure 3E,F). The CM significantly increased the mRNA levels of osteogenic osterix and Runx2 (Figure 3C,D).

#### 3.2.2. Effect of the Inhibition or Overexpression of miR-145 on the VSMC Phenotype

The antagomir transfection was effective and significantly reduced A7r5 miR-145 content by 79% (Scramble (control): 1.00 ± 0.12 R.U.; miR-145-antagomir (silencing): 0.21 ± 0.07 R.U.). 

In A7r5 cells exposed to non-CM, significant changes in Ca content were observed between the Scramble and miR-145 antagomir transfection; miR-145 silencing reduced α-actin gene expression by 27% (*p* < 0.01) and significantly increased osterix mRNA levels (*p* < 0.05) and Runx2 (*p* < 0.05). Under CM conditions, miR-145 silencing (transfection with antagomir) increased Ca content, despite no significant differences in α-actin, osterix, and Runx2 mRNA compared to VSMCs transfected with Scramble (Table 2).

The transfection with miR-145 mimic caused an 81-fold increase in miR-145 content (Scramble: 1.00 ± 0.12 R.U.; miR-145-mimic: 81.53 ± 33.15 R.U.). 

**Figure 4:**
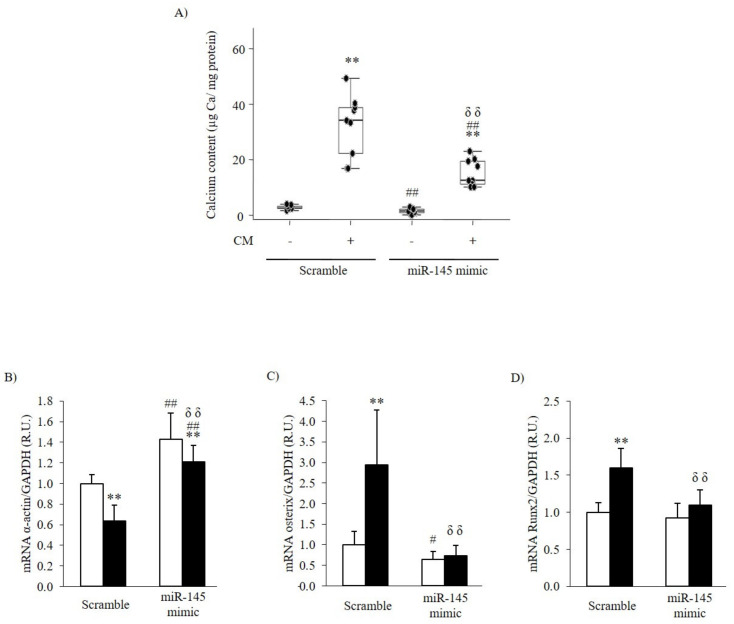
Overexpression of miR-145 in VSMC on calcification and osteogenic differentiation. (**A**) Ca deposition in A7r5 cells transfected with Scramble or miR-145 mimic and exposed to either non calcifying media (CM-) or calcifying media (CM+) for 4 days. Box plots represent median and interquartile range from three independent experiments performed in triplicate per experimental condition. (**B**) α–actin, (**C**) osterix and (**D**) Runx2 mRNA levels in A7r5 cells transfected with Scramble or miR-145 mimic and exposed to either non calcifying media (non-CM, white bars) or calcifying media (CM, black bars) for 4 days. Bars and error bars represent mean ± SD from three independent experiments performed in triplicate per experimental condition; R.U.: relative units. ** *p* < 0.01 vs. non calcifying media (CM-) within a transfection group; # *p* < 0.05 and ## *p* < 0.01 vs. Scramble + non calcifying media (Scramble + CM-); δδ *p* < 0.01 vs. Scramble + calcifying media (Scramble + CM+).

In non-CM, a significant reduction in Ca content between the Scramble and miR-145 mimic transfection was observed (Figure 4A). Under these conditions (white bars), miR-145 overexpression increased α-actin gene expression above the levels observed in Scramble transfected cells and significantly reduced osterix gene expression (Figure 4B,C); miR-145 overexpression had no effect on Runx2 mRNA (Figure 4D). 

Under CM, the overexpression of miR-145 attenuated by 44% the increase in total Ca deposition compared with that in Scramble transfected cells (*p* < 0.05) (Figure 4A). Under these conditions (black bars), the overexpression of miR-145 significantly prevented the decrease in α-actin gene expression and the increase in osterix and Runx2 gene expression (Figure 4B–D).

## 4. Discussion

In CKD patients, the prevention of uremia-induced vascular damage seems to be critical to attenuate the risk of cardiovascular morbidity and mortality [4]. Several studies have demonstrated the importance of the vitamin D hormonal system and some miRs to maintain a healthy and compliant arterial system, which is severely affected in patients with reduced renal function [27]. miR-145 plays an essential role in fully developed arteries to ensure a healthy differentiated VSMC contractile phenotype [8,9,10]. Indeed, the downregulation of miR-145 to almost undetectable levels concurs with proliferative vascular diseases and atherosclerotic lesions [9,10]. 

The present experimental in vivo and in vitro study addresses the importance of uremia in reducing miR-145 and its impact on the reduction in VSMC contractile phenotype, and explores the likely beneficial effect of the vitamin D hormonal system in the upregulation of miR-145 at improving the vascular phenotype. 

In CKD patients, the increased de-differentiation of VSMC to osteoblast-like cells, induced directly by high serum P [28] or inflammation-driven oxidative stress [29,30], is a major contributor to their higher propensity for Ca deposition in the medial artery layer that markedly enhances their risk of cardiovascular mortality [2,4,31]. Since uremia and high serum P cause aortic miR-145 reductions [8,14] and miR-145 is implicated in the regulation of the expression of contractile markers [10,11] and directly targets the osterix gene in osteoblasts [32], one of the goals of this study was to evaluate whether vascular miR-145 reductions could provide a non-traditional predictor of VSMC osteogenic differentiation and vascular calcification.

The contribution of uremia-induced reductions in aortic miR-145 content to prompt osteogenic differentiation was evaluated using the FVB/N mouse, a strain that develops secondary hyperparathyroidism after two months of renal mass reduction [33].

In our in vivo study, after 14 weeks of the induction of 75% renal mass reduction and the development of mild uremia with increases in BUN, PTH and renal levels of ADAM17 (an enzyme that increases in kidney disease of all etiologies) [23], no changes in serum Ca or P levels were observed. Aortic miR-145 decreased by 80%, with a paralleled marked reduction in α-actin mRNA but did not significantly increase aortic Ca deposition. Only aortic Runx2, the earliest marker of VSMC osteogenic differentiation [34,35], increased significantly compared to that in sham operated controls. This elevation in aortic Runx2 reproduced the findings of a previous study [35]. 

Our ex vivo and in vitro studies in aortic rings from normal rats and in the aortic rat VSMC line A7r5, exposed exclusively to calcifying conditions for 4 days, corroborated the association between the increase in osteogenic differentiation genes and Ca deposition with a significant reduction in miR-145 content. The reduction in miR-145 was accompanied by a significant reduction in α-actin and increase in osterix and Runx2 gene expression. These results are in agreement with previous studies demonstrating that miR-145 regulates the expression of contractile phenotype markers in VSMC, such as α-actin, among others [10,11]. Regarding VSMC osteogenic differentiation, miR145 reductions not only directly target osterix [36], but also the transcription factor KLF4, which directly controls Runx2 [17,18,19].

Importantly, the silencing of miR-145 in A7r5 cells exposed to non-calcifying conditions was as effective, as the exposure to calcifying conditions at reducing α-actin and inducing osterix gene expression. Accordingly, the marked attenuation of calcification achieved by miR-145 overexpression in A7r5 cells exposed to calcifying conditions is associated directly with the prevention of both α-actin reductions and elevations in osterix mRNA.

Significantly, the silencing of miR-145 in A7r5 cells under calcifying conditions induced a 51% increase in Ca content despite no changes in osterix and α-actin gene expression compared to scramble transfected cells. Forced changes in miR-145 content in VSMC exposed to calcifying conditions support the indirect regulation of Runx2 gene expression by miR-145, probably through modulation of KLF4 expression, as previously demonstrated [17,18,19]. 

The reduction in VSMC miR-145 could also promote the release of pro-calcifying exosomes, identified mediators to propagate calcification signals [37] through the induction of Rab27a protein expression [38], an essential component of the endosomal sorting complex for exosome transport and release, a possibility that could not be tested herein due to the small size of mice aortas.

Vitamin D induction of miR-145 gene expression [7] led us to propose that treatment with vitamin D from week 8 to 14 after the induction of renal mass reduction could attenuate uremia-induced reductions in aortic miR-145 and, consequently, their effects on VSMC de-differentiation predisposing to the loss of the contractile phenotype towards osteogenic differentiation and Ca deposition. The pharmacological approach, with a combination of 25(OH)D_3_ and the 1,25D_3_ analog paricalcitol, was based on the efficacy of this combination to simultaneously correct nutritional vitamin D deficiency and synergize in the control of secondary hyperparathyroidism without changes in serum Ca and P levels in a hyperphosphatemic rat model with kidney failure [21].

Treatment of uremic mice with the combination of 25(OH)D_3_ + paricalcitol resulted in PTH over-suppression, hypercalcemia and hyperphosphatemia, commonly occurring with excessive vitamin D therapy in advanced human CKD stages. Despite these severe systemic calcifying conditions, vitamin D therapy prevented aortic miR-145 reductions. Vitamin D-mediated maintenance of aortic miR-145 content was associated with a 50% attenuation of uremia-induced reduction in α-actin and prevention of the increase in Runx2. Consequently, despite having an excess of serum Ca and P, as well as over-suppression of PTH, the aortic Ca content did not increase.

Taken together, these findings suggest that the prevention of aortic miR-145 reduction and their impact on vascular injury could have partly contributed to the improved survival rates observed in hemodialysis patients receiving paricalcitol, beyond its efficacy to suppress PTH [39]. The increase in arterial miR-145 by vitamin D treatment suggests that part of its antiproliferative actions could involve the maintenance of VSMC miR-145 levels.

## 5. Conclusions

In conclusion, in established CKD, vitamin D treatment attenuates uremia-induced reductions in aortic microRNA-145 and its associated vascular damage, despite hyperphosphatemia, by attenuating the loss of the contractile phenotype of VSMC and, consequently, the onset of osteogenic differentiation, predisposing to Ca deposition.

## Figures and Tables

**Table 1:** Serum biomarkers of bone and mineral metabolism. Values indicate Mean ± SD or Median (interquartile range). * *p* < 0.05 and *** *p* < 0.001 vs. Sham; ^#^ *p* < 0.05, ^##^ *p* < 0.01 and ^###^ *p* < 0.001 vs. NX group.

	Sham (*n* = 10)	NX (*n* = 10)	NX + 25(OH)D_3_ + Paricalcitol (*n* = 10)
BUN (mg/dL)	26.20 ± 6.78	51.00 ± 10.27 ***	52.00 ± 7.16
Calcium (mg/dL)	13.08 ± 0.89	13.09 ± 0.40	14.81 ± 1.10 ^###^
Phosphate (mg/dL)	9.94 ± 1.16	9.52 ± 2.67	10.28 ± 1.47 ^#^
PTH (pg/mL)	190.00 ± 89.79	399.70 ± 292.21 *	50.80 ± 23.98 ^##^
FGF23 (ng/mL)	0.66 (0.62−0.71)	0.81 (0.67−0.83)	7.16 (4.21−11.43) ^###^

**Table 2:** Reductions of miR-145 in VSMC contribute to osteogenic differentiation and Ca deposition. Ca deposition, α–actin, osterix and Runx2 gene expression in A7r5 cells transfected with scramble or miR-145 antagomir and exposed to either non calcifying media (non-CM; 1 mM Ca, 1 mM P) or calcifying media (CM; 2 mM Ca, 3 mM P) for 4 days. Values indicate Mean ± SD or Median (interquartile range) from three independent experiments performed in triplicate per experimental condition; R.U.: relative units; ** *p* < 0.01 vs. non-CM within a transfection group; ^#^ *p* < 0.05 and ^##^ *p* < 0.01 vs. Scramble + Non CM; ^δδ^ *p* < 0.05 vs. Scramble + CM.

	Scramble		miR-145 Antagomir	
	Non-CM	CM	Non-CM	CM
Ca content (µg Ca/mg protein)	2.31 [2.20–3.02]	33.96 [22.05–38.72] **	3.65 [3.43–5.56] ^##^	50.90 [48.29–57.47] **^,##,δδ^
mRNA α-actin/GAPDH (R.U.)	1.00 ± 0.09	0.64 ± 0.15 **	0.73 ± 0.10 ^##^	0.59 ± 0.21
mRNA Osterix/GAPDH (R.U.)	1.00 ± 0.33	2.94 ± 1.34 **	1.73 ± 0.74 ^#^	2.61 ± 1.07
mRNA Runx2/GAPDH (R.U.)	1.00 ± 0.13	1.60 ± 0.26 **	1.15 ± 0.10 ^#^	1.76 ± 0.26 **

## Data Availability

The data underlying this article will be shared upon reasonable request to the corresponding author.
